# ROS-mediated cell death and phase separation in gynecological malignancies

**DOI:** 10.1186/s40001-025-02846-3

**Published:** 2025-07-05

**Authors:** Huabing Wei, Meifeng Xiong, Ling Min

**Affiliations:** 1 Department of Gynecology and Obstetrics, Leshan Women and Children Hospital, Leshan, China; 2 Department of Gynecology and Obstetrics, Development and Related Diseases of Women and Children Key Laboratory of Sichuan Province, Key Laboratory of Birth Defects and Related Diseases of Women and Children, West China Second Hospital, Sichuan University, Chengdu, China

**Keywords:** Reactive oxygen species, Oxidative stress, Phase separation, Cell death, Gynecological malignancies

## Abstract

Reactive oxygen species (ROS) are reactive products of cellular metabolism that, under physiological conditions, activate specific signaling pathways essential for cellular functions. Excessive accumulation of ROS overwhelms cellular antioxidant defenses, leading to functional damage or cell death. ROS-mediated cell death manifests in multiple forms, including apoptosis, ferroptosis, immunogenic cell death, pyroptosis, oxeiptosis, NETosis, and parthanatos. In gynecological malignancies, inducing ROS-mediated cell death is proposed as a therapeutic strategy. Furthermore, research indicates that excessive ROS promotes abnormal phase separation, resulting in functional damage to tumor cells. Therefore, ROS-mediated cell death and phase separation provide a new entry point for preventing tumorigenesis or treating gynecological malignancies. In this review, we summarize the mechanisms underlying ROS-mediated cell death and phase separation, and describe innovative strategies based on ROS that hold promise for treating gynecological malignancies. This review will also discuss controversial issues in tumor therapeutics, including the paradoxical roles of ROS manipulation. This will offer new insights into the management of gynecological malignancies, providing theoretical support for clinical practice.

## Introduction

Gynecological malignancies, mainly including cervical cancer, ovarian cancer, and endometrial cancer, are the leading cause of mortality among women worldwide [1]. Epidemiological data reveal the burden of gynecological cancers (https://seer.cancer.gov). Each year, there are more than 570,000 new cases and 300,000 deaths from cervical cancer, approximately 225,500 new cases and 140,200 deaths from ovarian cancer. In addition, there are more than 63,230 new cases and 11,350 deaths from endometrial cancer in the United States each year [2]. Hence, urgent efforts are needed to develop innovative therapeutic strategies and drugs for gynecological malignancies.

It is now evident that ROS are important for regulating signaling pathways involved in cell growth and differentiation under physiological conditions [3]. While moderate levels of ROS contribute to various biological processes, the overproduction of ROS can lead to cell dysfunction and death [4]. In addition, excessive ROS can undermine phase separation, a process where biomolecules condense to form membrane-less organelles [5]. These findings suggest that ROS play a significant role in phase separation and cell death, further indicating that inducing ROS-mediated phase separation and cell death represent new therapeutic strategies for treating gynecological malignancies.

Herein, we comprehensively summarize the underlying mechanisms by which ROS-mediated phase separation and multiple forms of regulated cell death. By discussing these molecular events, this review provides foundational perspectives for future studies of ROS-based therapeutic strategies and aims to guide the effective treatments for gynecological malignancies.

## ROS-mediated cell death in gynecological malignancies

### ROS and apoptosis in gynecological malignancies

Apoptosis is a well-defined mode of programmed cell death that converges on caspase activation [6]. It is characterized by cell shrinkage, DNA fragmentation, and the formation of membrane-surrounded apoptotic bodies [7]. As upstream regulators, ROS integrate signals that converge on mitochondrial dysfunction and death receptor activation, amplifying the apoptotic signaling cascade through both intrinsic and extrinsic pathways.

**Fig. 1:**
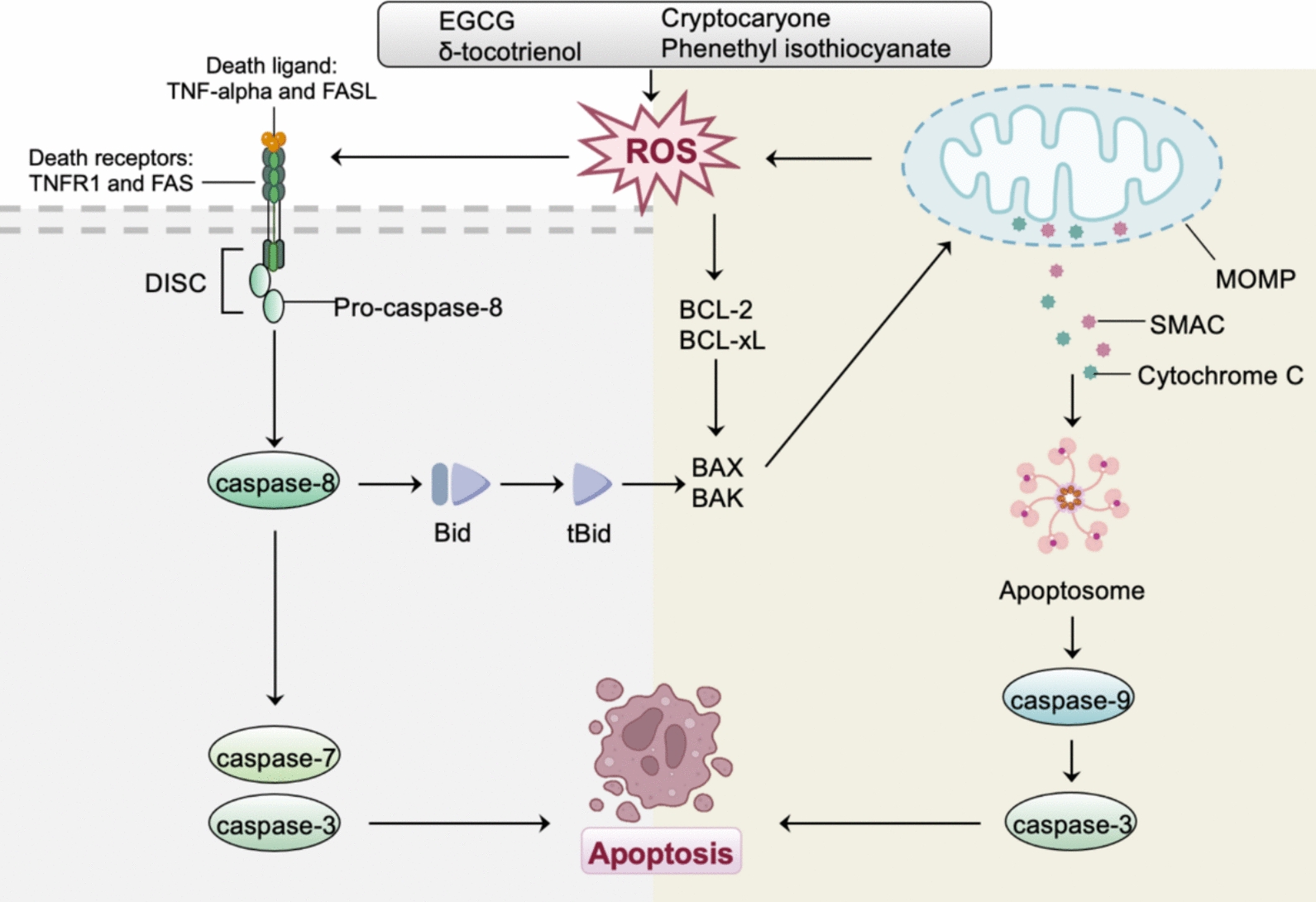
ROS-mediated apoptosis in gynecological malignancies. ROS promote apoptosis by inducing mitochondrial outer membrane permeabilization (MOMP), activating pro-apoptotic Bcl-2 family proteins (e.g., BAX and BAK), inhibiting anti-apoptotic proteins (e.g., Bcl-2 and Bcl-xL), and enhancing death receptor signaling. In gynecological malignancies, δ-tocotrienol, cryptocaryone, phenethyl isothiocyanate, and (−)-Epigallocatechin-3-gallate (EGCG) can induce apoptosis by stimulating the generation of ROS, indicating that ROS-mediated apoptosis has therapeutic significance

In gynecological cancers, ROS activate the JNK pathway, induce DNA damage, and disrupt mitochondrial function, providing a prerequisite for promoting apoptosis of tumor cells (Fig. 1). Furthermore, during chemotherapy, uncontrolled surges of ROS worsen mitochondrial dysfunction, forming a self-perpetuating vicious cycle that intensifies apoptosis [8]. Specifically, δ-tocotrienol induces apoptosis and sensitizes ovarian cancer cells to cisplatin through the production of mitochondrial ROS [9]. Cryptocaryone, a natural product, triggers ovarian cancer cells apoptosis by stimulating the generation of ROS and mitochondrial superoxide [10]. In cervical cancer cells, phenethyl isothiocyanate increases the production of intracellular ROS in a dose-dependent manner and induces ROS-mediated apoptosis, evidenced by the activation of caspase-3. In endometrial cancer, (−)-Epigallocatechin-3-gallate (EGCG) inhibits cellular proliferation via inhibiting ERK activation and induces apoptosis via ROS generation and p38 activation [11].

### ROS and ferroptosis in gynecological malignancies

**Fig. 2:**
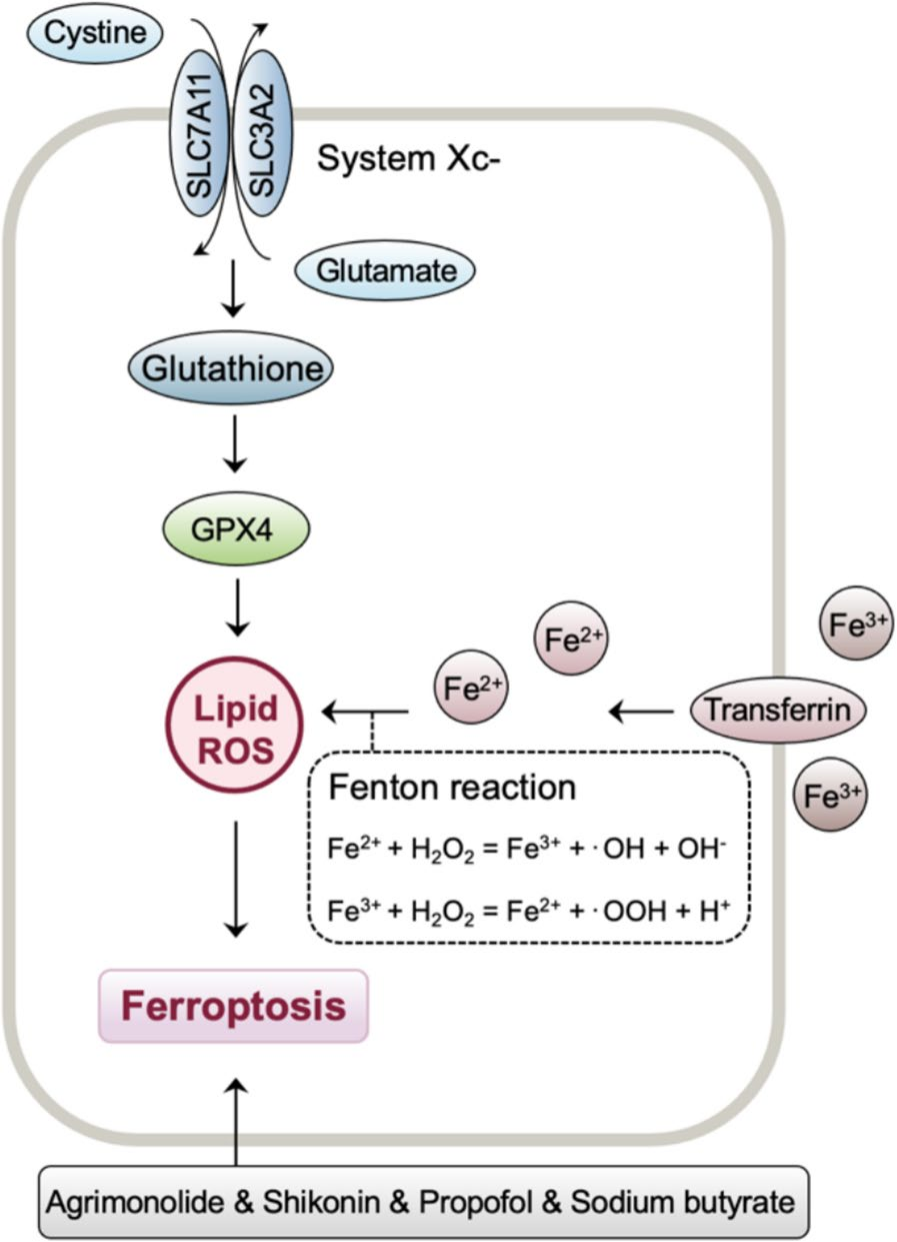
ROS-mediated ferroptosis in gynecological malignancies. The production of ROS through the iron-dependent Fenton reaction drives ferroptosis by promoting lipid peroxidation, and antioxidant systems, such as glutathione (GSH) and glutathione peroxidase 4 (GPX4), counteract iron-induced lipid ROS. In gynecological malignancies, agrimonolide, shikonin, propofol and sodium butyrate can induce ROS-mediated ferroptosis

The concept of ferroptosis was proposed by Dr. Brent R. Stockwell in 2012 to describe an iron-dependent, lipid peroxidation-driven cell death mode (Fig. 2) [12]. Research indicates that the disruption of intracellular iron homeostasis and lipid peroxide metabolism are the main factors driving ferroptosis [13, 14]. Furthermore, directly downregulating the activity of glutathione peroxidase 4 (GPX4) or indirectly inhibiting the activity of GPX4 by depleting the level of intracellular glutathione (GSH) and inhibiting the function of the cystine (Cys)/glutamic acid (Glu) antiporter system (System Xc-) can diminish the total antioxidant capacity and promote the accumulation of ROS, thereby inducing ferroptosis [15].

In ovarian cancer, agrimonolide inhibits cell proliferation and migration in a dose-dependent manner while promoting ferroptosis through increasing ROS and iron levels [16]. Moreover, the combination of shikonin and cisplatin synergistically induces ferroptosis in cisplatin-resistant tumor cells, evidenced by increased ROS, lipid peroxidation, and Fe^2+^ levels [17]. Similarly, propofol augments ferroptosis induced by paclitaxel in cervical cancer cells by increasing the levels of ROS and concentrations of Fe^2+^ [18]. In addition, sodium butyrate induces ferroptosis in endometrial cancer cells through the RBM3/SLC7A11 axis, accompanied by increased ROS and lipid peroxidation levels [18].

### ROS and immunogenic cell death in gynecological malignancies

Immunogenic cell death (ICD) is a newly defined form of regulated cell death that activates adaptive immune responses against tumor-specific antigens [19]. ICD is characterized by the release of damage-associated molecular patterns (DAMPs) from dying cells, including calreticulin (CRT), high mobility group box 1 (HMGB1), and adenosine triphosphate (ATP) [20]. Through the recognition by pattern recognition receptors (PRRs), DAMPs enhance the immunogenicity of tumor cells, and ultimately induce ICD [21].

**Fig. 3:**
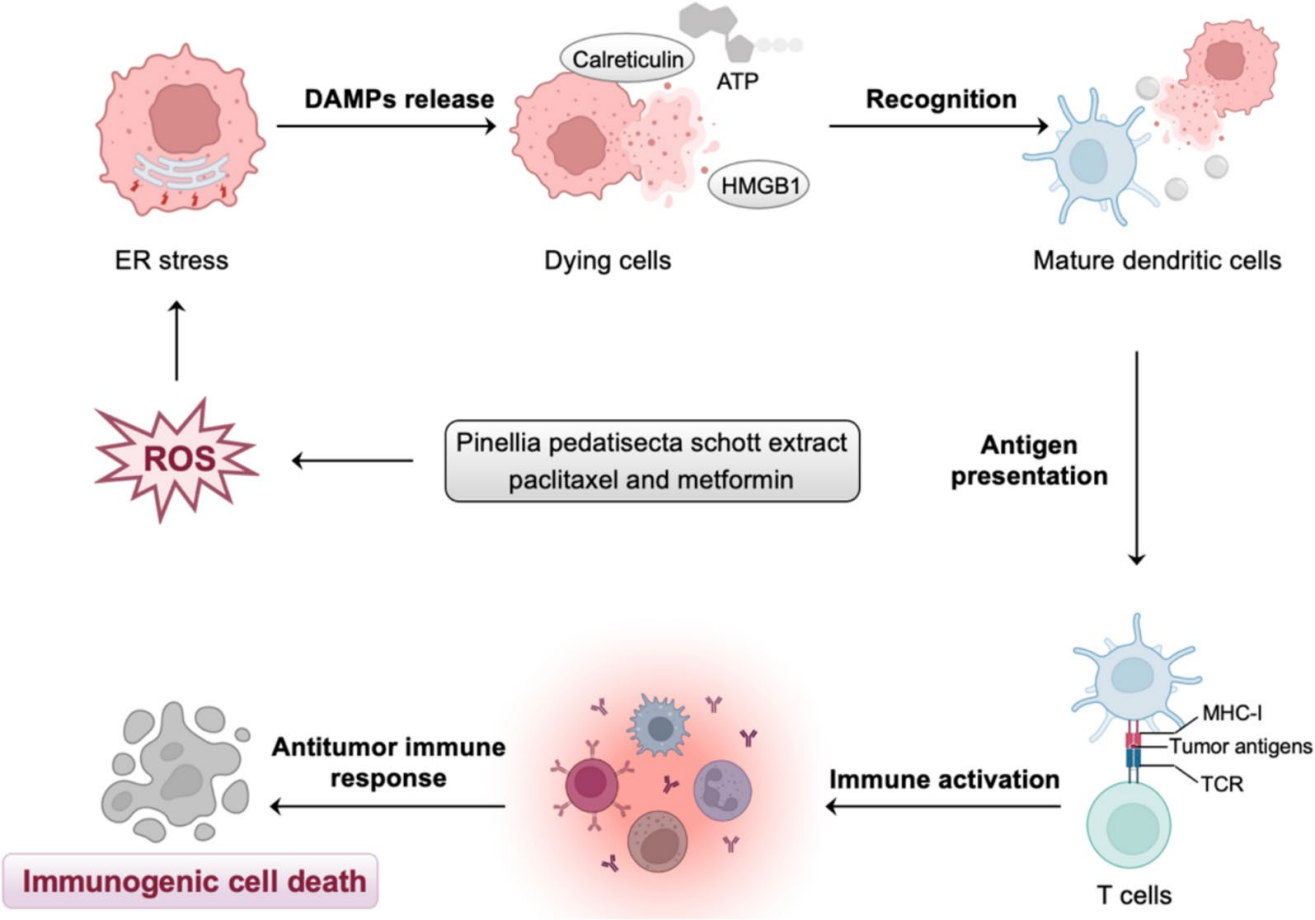
ROS-mediated immunogenic cell death (ICD) in gynecological malignancies. High levels of intracellular ROS induce ICD by triggering endoplasmic reticulum (ER) stress and the release of damage-associated molecular patterns (DAMPs). In gynecological malignancies, pinellia pedatisecta schott extract (PE), paclitaxel, and metformin can induce excessive production of ROS, thus inducing the immunogenic death of cancer cells

It has been proposed that increased levels of intracellular ROS are important for triggering ICD by potentiating danger signaling pathways, which facilitate the translocation of DAMPs to the extracellular milieu [22] (Fig. 3). In cervical cancer, pinellia pedatisecta schott extract (PE) increases ROS levels by impeding upstream antioxidant mechanisms, thus inducing the immunogenic death of cancer cells [23]. Furthermore, combining PE with cisplatin enhances the immunogenic effect of cisplatin, suggesting that the synergistic use of PE and cisplatin holds the potential for improving immunochemotherapy in cancer therapy [23]. In ovarian cancer, it has been shown that paclitaxel and metformin can induce excessive production of ROS and reduce antioxidant capacity, resulting in cellular oxidative damage [8, 24]. Recent studies further suggest that paclitaxel and metformin can induce immunogenic death of ovarian cancer cells [25, 26].

### ROS and pyroptosis in gynecological malignancies

**Fig. 4:**
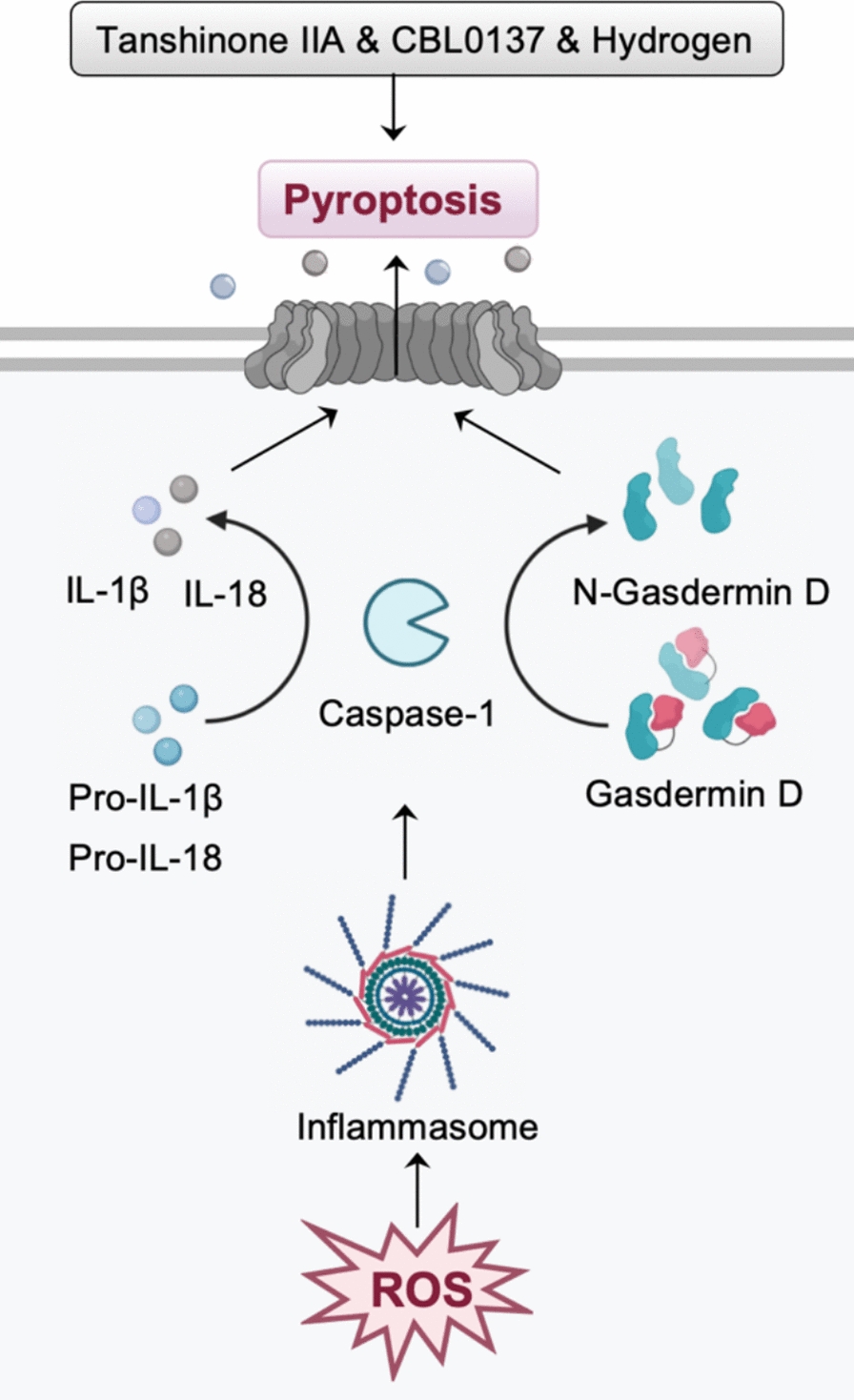
Pyroptosis is programmed inflammatory cell death that can be triggered by ROS, which activate the inflammasome and upregulate proinflammatory cytokines (e.g., IL-1β and IL-18). Excessive ROS promote the activation of the inflammasome, maturation of caspase-1, and cleavage of gasdermin D, resulting in pore formation and inflammasome-dependent pyroptosis. In gynecological malignancies, tanshinone IIA, small molecule inhibitor CBL0137, and hydrogen can induce inflammasome-mediated pyroptotic pathways

Pyroptosis is a form of programmed inflammatory cell death characterized by the activation of inflammasomes and inflammation-associated caspases [27]. The best-characterized pyroptosis involves multiple stages: the assembly and activation of the inflammasome, the cleavage of gasdermin D, and the formation of membrane pores [28]. ROS act as upstream signals for the activation of the NLRP3 inflammasome by upregulating the expression of components, including NLRP3, pro-caspase-1, and pro-IL-1β, thereby promoting the assembly and activation of the NLRP3 inflammasome (Fig. 4) [29].

In gynecological malignancies, hydrogen can inhibit endometrial cancer by ROS/NLRP3/caspase-1/gasdermin D-mediated pyroptotic pathway [30]. Tanshinone IIA can promote pyroptosis in cervical cancer cells and exert anticancer activity by regulating the mir-145/gasdermin D signaling [31]. CBL0137, small molecule inhibitor, can activate ROS/BAX signaling to promote gasdermin E-dependent pyroptosis in ovarian cancer cells [32]. Furthermore, it has been found that CBL0137 inactivates the chromatin remodeling complex and decreases the transcription of antioxidants, leading to elevated levels of ROS [32].

### ROS and other cell death

**Fig. 5:**
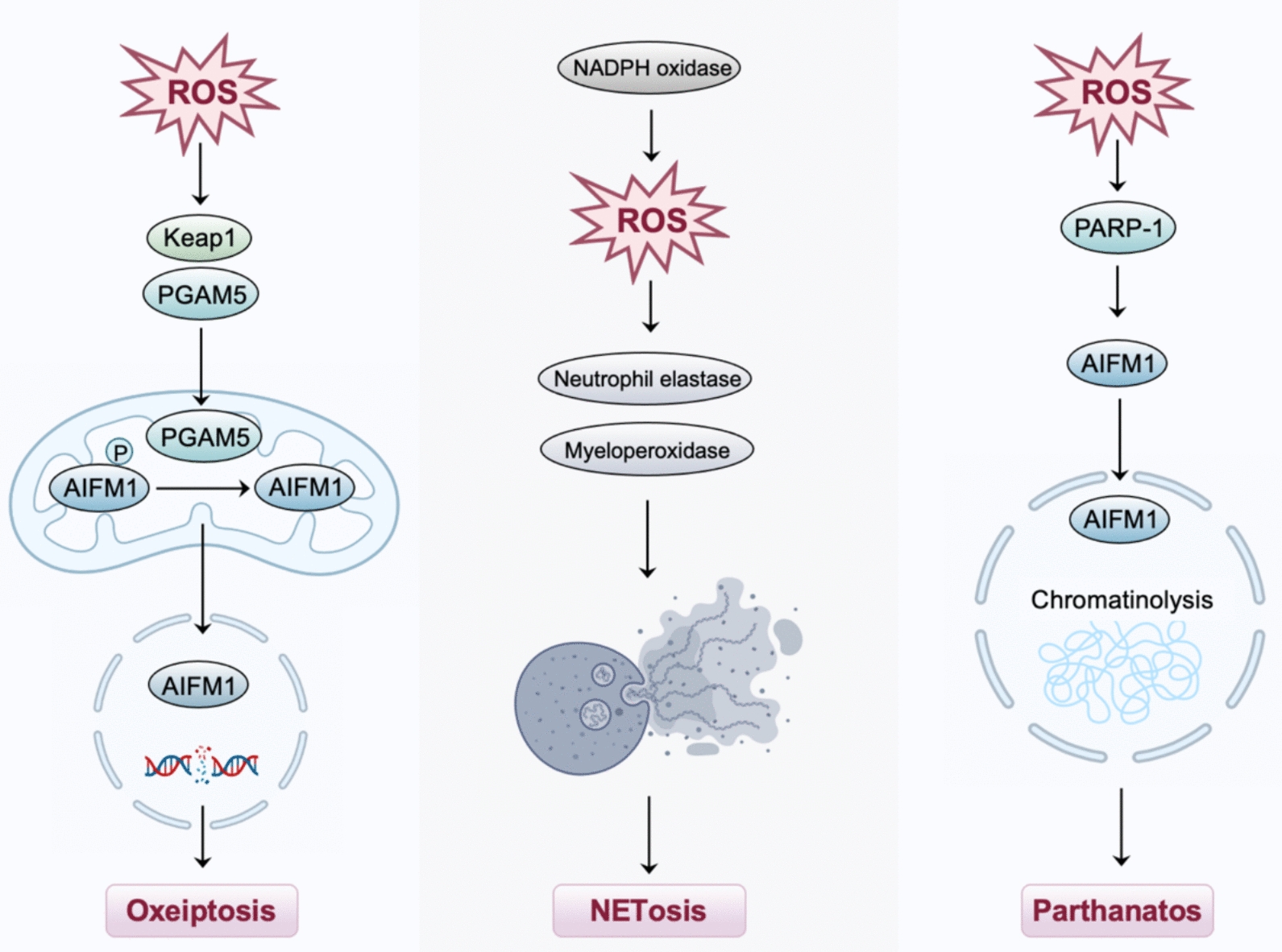
Role of ROS in oxeiptosis, NETosis, and parthanatos

In 2018, Holze et al*.* first proposed a programmed cell death mode closely related to ROS, known as oxeiptosis [33]. Oxeiptosis is a caspase-independent non-inflammatory cell death pathway characterized by the massive accumulation of ROS that activates the Keap1-phosphoglycerate mutase family member 5 (PGAM5)-apoptosis inducing factor mitochondria associated 1 (AIFM1) axis [33, 34] (Fig. 5). Specifically, the process is initiated by the pathological accumulation of ROS in cells and mediated by the ROS sensor Keap1, phosphatase PGAM5, and pro-apoptotic factor AIFM1 signaling cascade [33, 35].

Netotic cell death (NETosis) is a form of programmed cell death in which neutrophils trigger neutrophil extracellular traps (NETs) upon stimulation [36]. At the molecular level, NETosis is a dynamic process that involves the production of ROS mediated by NADPH oxidase, the release and translocation of myeloperoxidase, the oxidative activation of neutrophil elastase, the trafficking of N-GSDMD from the cytosol to the nucleus, and the autophagic pathway [37, 38].

Parthanatos is a poly ADP-ribose polymerase-1 (PARP-1)-dependent programmed cell death, which is activated by ROS-induced DNA damage [39]. Exposure to ROS stimulates PARP1 activation and subsequent AIFM1 nuclear translocation in glioma cells, whereas antioxidant interventions have been shown to mitigate ROS-induced parthanatos [40].

Despite limited mechanistic insight into how ROS regulate oxeiptosis, parthanatos, and NETosis, these cell death modalities warrant further investigation as potential strategies in the treatment of gynecological malignancies.

## ROS-mediated phase separation in gynecological malignancies

### The concept of phase separation

Initially, phase separation is a concept of physical chemistry, which refers to the formation of distinct phases within a system due to differences in the interactions between different components. These phases can be obviously distinguished in space and morphology. For example, the oil droplets floating on the water surface that can be seen everywhere are a typical liquid–liquid-phase separation (LLPS) phenomenon. In 2009, the concept of “phase separation” was introduced into biological research, explicitly referring to the compartmentalization and concentration of biomacromolecules in cells [41]. It explains the rapid and reversible formation of dynamic membraneless organelles in cells, including the microRNA-induced silencing complex (miRISC), stress granules, and P granules [41].

### The regulation of ROS in phase separation

**Fig. 6:**
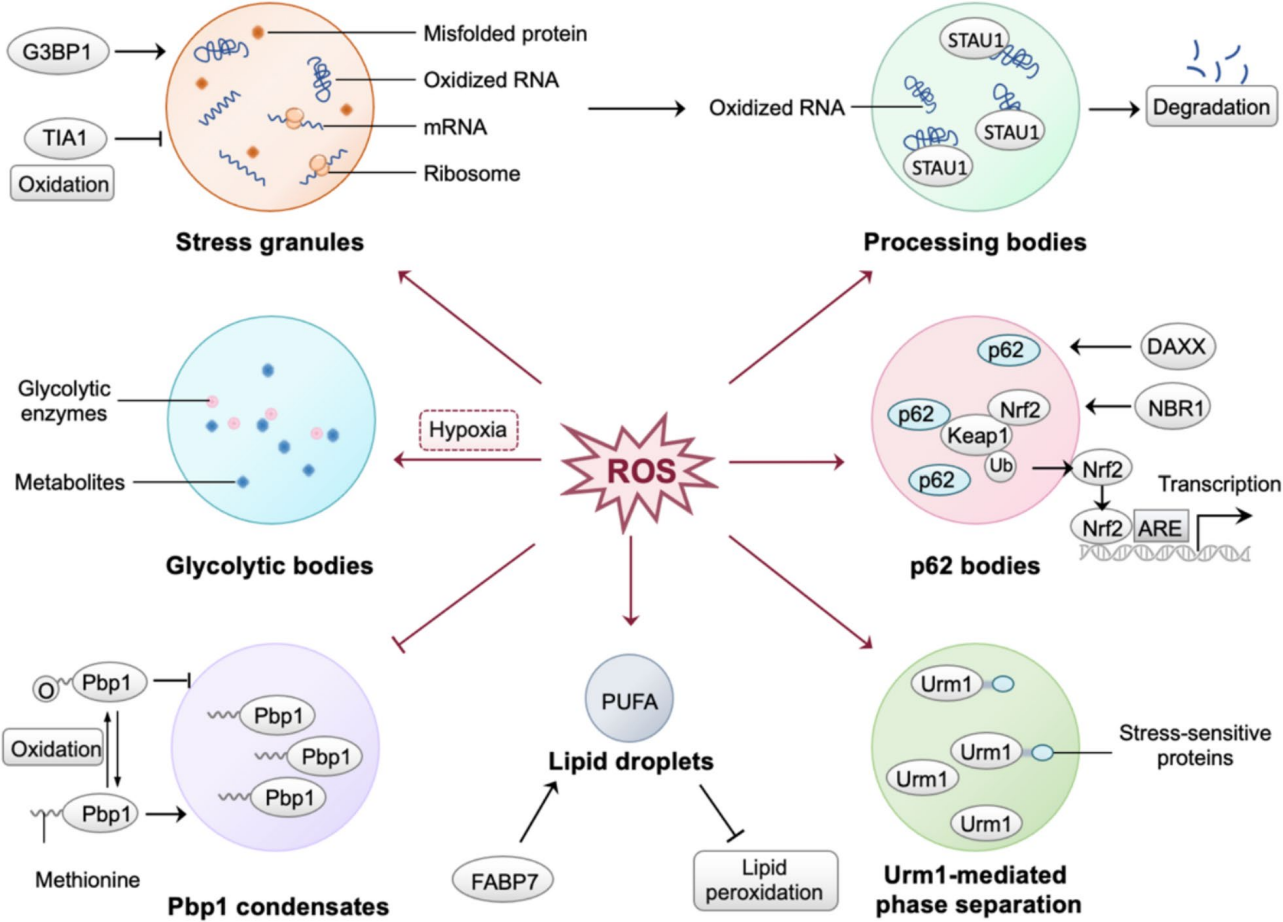
Illustration of ROS-mediated phase separation. (1) inducing the formation of stress granules, which sequester ribosomes and mRNA; (2) promoting the formation of processing bodies (P-bodies), which receive oxidized RNA from stress granules by staufen double-stranded RNA binding protein 1 (STAU1) for degradation; (3) inducing the formation of glycolytic bodies under hypoxic conditions, concentrating glycolytic enzymes and metabolites to enhance metabolic efficiency; (4) facilitating p62 bodies to sequester Keap1 and release Nrf2 through death domain associated protein (DAXX) and autophagy receptor neighbor of BRCA1 gene 1 (NBR1), driving the expression of antioxidant genes; (5) stimulating the oxidation of methionine in penicillin-binding protein 1 (Pbp1), leading to the disassembly of condensates; (6) inducing the formation of lipid droplets through fatty acid binding protein 7 (FABP7), which compartmentalize polyunsaturated fatty acids (PUFA) and prevent lipotoxicity; (7) triggering the stress-dependent phase separation mediated by Urm1

Based on the current study, Chen et al*.* proposed that ROS regulate phase separation through six mechanisms, allowing cells to respond to oxidative stress by organizing proteins and other molecules into distinct membrane-less compartments [42]. In this section, we briefly describe ROS-mediated phase separation according to the above six mechanisms and supplement the stress-dependent phase separation mediated by Urm1 (Fig. 6).

#### Stress granules

Stress granules (SGs) are dynamic, reversible assemblies of mRNA and proteins formed through liquid–liquid-phase separation in response to stresses [43]. In this process, G3BP1 serves as a molecular switch that initiates RNA-dependent LLPS in response to increased intracellular free RNA concentrations [44]. Excessive ROS can suppress the formation of SGs by oxidizing the Cys36 residue of the SGs-nucleating protein T-cell intracellular antigen 1 (TIA1), resulting in the promotion of apoptosis [45].

#### Processing bodies

Oxidative stress, particularly ROS, promotes the formation of processing bodies (P-bodies), which receive oxidized RNA from SGs for degradation [46]. Specifically, P-bodies transfer oxidized RNA in SGs through staufen double-stranded RNA binding protein 1 (STAU1) and function as membrane-less organelles for degradation [47]. Overall, SGs and P-bodies are coexisting liquid phases that compartmentalize oxidation products, providing a system for cellular quality control [42].

#### Glycolytic bodies

Hypoxic conditions drive cells to form glycolytic bodies (G bodies), which concentrate glycolytic enzymes and metabolites, enhancing metabolic efficiency under low oxygen levels [48]. This compartmentalization enables cells to better cope with nutrient deprivation, a common feature of cancerous tissues.

#### P62 bodies

As an autophagic receptor, p62/sequestosome-1 (SQSTM1) binds to ubiquitinated proteins and undergoes phase separation to form membrane-less organelles, p62 bodies [49]. When intracellular ROS accumulation is excessive, p62 bodies sequester Keap1 and release Nrf2, which binds to ARE and drives the expression of antioxidant genes [50]. This process is regulated by death domain associated protein (DAXX) and autophagy receptor neighbor of BRCA1 gene 1 (NBR1), which promote p62 condensation and enhance the cellular antioxidant response [49, 51, 52].

#### Penicillin-binding protein 1 condensates

ROS, particularly H₂O₂, mediate phase separation by reversible oxidation of cysteine residues and methionine residues in intrinsically disordered regions of proteins [53, 54]. For instance, penicillin-binding protein 1 (Pbp1) forms condensates in response to changes in mitochondrial respiration, while the oxidation of methionine in Pbp1 induced by H₂O₂ leads to the disassembly of condensates [54]. The reversibility of this process enables cells to respond rapidly to ROS and adapt to oxidative stress.

#### Lipid droplets

During oxidative stress, ROS stimulate the formation of lipid droplets, which compartmentalize polyunsaturated fatty acids (PUFA) and prevent lipotoxicity and membrane damage [55]. In glioma cells, fatty acid binding protein 7 (FABP7) internalizes long-chain fatty acids to promote lipid droplet formation, while in astrocytes with FABP7 knockout, the ability of ROS to induce lipid droplet formation is diminished [56].

#### Stress-dependent phase separation mediated by Urm1

Stress, including tert-butyl peroxide stimulation, can trigger the self-aggregation of Urm1 and its interaction with target proteins and the Urm1-conjugating enzyme Uba4, thereby facilitating the phase separation [57]. In this process, stress-sensitive proteins modified by Urm1 are sequestered into SGs and nuclear condensates. In contrast, cells lacking Urm1 have defects in the phase separation process and demonstrate decreased stress tolerance.

### Implications of phase separation in gynecological malignancies

Phase separation dynamically organizes molecular components into compartmentalized structures to regulate multiple biological processes, including gene transcription, signal transduction, and cellular homeostasis [58]. Recent studies have shown that abnormal phase separation is involved in the dysregulation of physiological processes and contributes to tumor development [59]. In ovarian cancer and endometrial cancer, phase separation-related genes have been identified as biomarkers for disease prognosis. Insights from other cancer types suggest that phase separation-associated genes may serve as diagnostic or therapeutic targets. For example, BRD4, a key mediator of super-enhancer formation via phase separation, has been targeted by inhibitors in pancreatic cancer [60]. Similarly, PGAM1 undergoes phase separation to promote metabolic reprogramming in breast cancer, representing a potential metabolic vulnerability [61]. These findings suggest that analogous mechanisms may exist in gynecological cancer and warrant further investigation.

Consistently, ROS-mediated phase separation is a finely tuned mechanism that enables cells to concentrate essential proteins and enzymes in specific compartments, thus enhancing the efficiency of cellular processes, such as metabolic regulation and antioxidant defense [42]. Therefore, excessive ROS-induced disruptions in phase separation may contribute to the development of diseases, including gynecological malignancies. Disconsolately, there is no convincing evidence directly linking ROS-mediated phase separation to gynecological malignancies, and further research is necessary to establish this connection.

## ROS-based therapeutic strategies and phase separation-based drugs

### Inducing the generation of ROS

#### Photodynamic therapy

PDT is a clinically approved, non-invasive cancer therapy based on photochemical reactions [62]. During PDT, photosensitizers (PSs) are activated by light of a specific wavelength to produce cytotoxic ROS [63]. Upon activation by light, photosensitizers undergo two simultaneous photochemical reaction pathways: type I generates free radical ROS (superoxide and hydroxyl radicals) through electron transfer, while type II produces cytotoxic singlet oxygen through energy transfer [64]. Furthermore, numerous studies support that PDT can be combined with photothermal therapy (PTT), chemotherapy, or immunotherapy to improve the therapeutic effects of gynecological malignancies [62, 65].

In cancer therapy, PSs are localized in various cellular organelles, including mitochondria, the endoplasmic reticulum, lysosomes, and the plasma membrane, allowing the generated cytotoxic ROS to induce cell death through multiple pathways [63]. As exemplified above, PDT can activate photosensitizers in the ER to induce ROS and calreticulin exposure to release proinflammatory cytokines and DAMPs, leading to the ICD of tumor cells [66]. Furthermore, numerous studies support that PDT can be combined with photothermal therapy (PTT), chemotherapy, or immunotherapy to improve the therapeutic effects of gynecological malignancies [62, 65].

#### Sonodynamic therapy

SDT activates sonosensitizers through low-intensity ultrasound to enhance the generation of ROS in cancer cells, ultimately leading to tumor destruction [67]. Recently, SDT has attracted widespread attention due to its non-invasive, non-ionizing radiation and tissue penetration [68]. The main mechanisms of SDT involve the generation of ROS through cavitation-activated sonosensitizers, mechanical damage to cells caused by acoustic pressure, and thermal damage from ultrasonic sonication [69, 70]. Specifically, the cavitation effect triggers ROS production by rupturing bubbles, generating high temperatures and pressures that decompose water into hydroxyl radicals.

SDT effectively induces ROS-mediated cell death, especially ICD, which plays an important role in systemic anti-tumor immune responses [71, 72]. For example, engineering nanoplatforms camouflaged with macrophage membranes for sonodynamic therapy in ovarian cancer, which enhances macrophage phagocytosis, reprograms tumor-associated macrophages (TAMs) to the M1 phenotype, and improves the immunosuppressive tumor microenvironment to increase antitumor efficacy [73]. In gynecological malignancies, recent studies have shown that SDT can be combined with phototherapy or chemotherapy, offering a promising strategy for antitumor immunity and integrated diagnosis and treatment [74–76].

#### Chemodynamic therapy

CDT refers to the introduction of iron-based nanomaterials or other transition metals as the catalyst to trigger Fenton reactions, thereby in situ catalyzing H_2_O_2_ to produce cytotoxic ROS and kill cancer cells. Compared with photodynamic or sonodynamic therapy, CDT can be performed independently without external energy input and oxygen, thus avoiding the disadvantages of limited tissue penetration depth and hypoxia in the tumor microenvironment [77]. Moreover, CDT offers advantages, such as higher catalytic efficiency, lower side effects, and fewer equipment requirements, making it a promising clinical therapy [78].

To enhance the efficacy of CDT in cancer therapy, combination therapies involving CDT and other modalities, such as SDT and PDT, have been developed [79, 80]. For example, biodegradable nanocomposites for delivery of doxorubicin overcome drug resistance by promoting oxidative stress and enhance SDT, CDT, and chemotherapy [79]. Moreover, a photodynamic–chemodynamic cascade strategy was developed using an ROS-responsive polymersome for drug delivery, demonstrating enhanced antitumor effects and offering a promising approach for combination therapy [80].

### Phase separation-based drug development

In addition to ROS-based therapeutic strategies, developing drugs that target phase separation holds promise as a novel strategy for related diseases. Phase separation can be regulated by targeting intrinsically disordered regions or modulating conformational dynamics [81, 82]. Given the complexity of the phase separation process, the types of molecules that regulate the phase separation process are diverse, including small molecules, antibodies, and synthetic peptides [83]. For example, an interfering peptide designed to target the dimerization domain effectively disrupts the phase separation of the SARS-CoV-2 virus nucleocapsid protein, demonstrating inhibition of viral replication and restoration of innate antiviral immunity [84].

In general, the design and development of phase separation-related drugs is still in its infancy, and drug design based on intrinsically disordered regions of phase separation protein is a possible way to develop phase separation regulators.

## Prospects and challenges of ROS-based therapy

The temporospatial dynamic behaviors of ROS in the biological milieu can partly explain their controversies in cancer therap. ROS have a “double-edged sword” role in cancer biology, functioning as both promoters of tumor development and inducers of tumor cell death [85]. Accordingly, the challenge lies in precisely modulating ROS levels to selectively enhance anti-tumor effects while minimizing pro-tumor properties [86]. Excitingly, recent advancements in nanomedicine have promoted the development of nanomaterials with ROS-regulating properties, enabling precise control of ROS dynamics in vivo and providing new avenues for cancer therapy.

Ensuring the specificity of ROS regulation to target tumor cells while minimizing damage to normal cells poses a challenge in clinical applications [87]. In general, tumor cells possess higher ROS levels and an altered redox status due to genetic mutations and metabolic reprogramming, making them more susceptible to ROS-induced damage [88]. However, excessive ROS may also alter the fate of normal cells, leading to cytotoxicity and systemic side effects [89]. Accordingly, research should focus on pinpointing specific targets in tumor cells that can be selectively modulated through ROS, without affecting normal cells [90]. In addition, the application of advanced delivery systems is expected to improve the therapeutic index of ROS-mediated strategies, making ROS-based cancer therapy more precise in clinical practice [87].

Moreover, ROS modulators can be strategically combined with other therapeutic approaches, such as chemotherapy or immunotherapy, to enhance tumor sensitivity and maximize therapeutic effects [91, 92]. For instance, ROS-induced oxidative stress has been reported to promote the release of DAMPs, thereby eliciting a stronger antitumor immune response [91, 92]. Similarly, ROS can enhance the efficacy of radiotherapy by amplifying DNA damage and oxidative stress in tumor cells, thereby increasing radiosensitivity [91, 92].

ROS-mediated phase separation is an emerging concept with therapeutic promise, particularly in cancer and neurodegenerative diseases [42, 93]. Currently, there are several strategies and directions for cancer therapy by regulating phase separation, including (1) regulating the concentration of phase separation core proteins; (2) interfering with phase separation-related binding molecules, such as RNA and molecular chaperones; (3) controlling the environmental conditions for phase separation; (4) interfering with the regulatory parameters required for phase separation, such as membrane surfaces. However, the development of compounds that selectively target pathological phase separation without affecting physiological condensates is still in its early stages. The integration of live-cell imaging, high-throughput screening technologies, and multi-omics approaches will be essential for advancing ROS modulators toward therapeutic applications.

## Conclusion

In summary, rational manipulation of ROS has emerged as an opportunity to reshape the field of cancer therapeutics. The interaction between ROS, cell death pathways, and phase separation unveils a multifaceted yet promising landscape, offering practical and personalized options for treating gynecological malignancies. Furthermore, the use of ROS in combination would be clinically advantageous in enhancing the sensitivity of gynecological malignancies to chemotherapy. In the future, ROS modulators and their combination should be regarded as a supplement to precision medicine and a new paradigm for cancer therapy.

## Data Availability

No datasets were generated or analysed during the current study.
